# Heterologous Expression and In Vivo Functional Analysis of OsTPS2 in Yeast, Tobacco, and Rice

**DOI:** 10.3390/plants15111614

**Published:** 2026-05-25

**Authors:** Hua Li, Saiwen Li, Xiaojia Zhang, Yanping Luo

**Affiliations:** 1 School of Tropical Agriculture and Forestry, Hainan University, Danzhou 571700, China; 2 School of Ecology, Hainan University, Haikou 570228, China

**Keywords:** OsTPS2, neocembrene, OsTPS28, casbene-type diterpene, *Oryza sativa*

## Abstract

Casbene-type macrocyclic diterpenes possess a range of biological activities, with casbene and neocembrene serving as key precursor compounds. While there is an abundance of research on casbene synthase, in-depth studies in vivo on neocembrene synthase are relatively scarce. This study presents the first functional validation in vivo of OsTPS2 as a primary neocembrene synthase in rice, as demonstrated through both transient tobacco expression and yeast-induced expression systems. Comparative analysis with the OsTPS28 gene revealed that OsTPS2 produces a higher content of neocembrene. Furthermore, the overexpression of OsTPS2 in rice resulted in an increased neocembrene content within the leaves. Additionally, both OsTPS2 and OsTPS28 were found to induce the synthesis of geranylgeraniol in yeast. Geranylgeraniol exhibits antifungal properties against *Rhizoctonia solani* and *Phomopsis* sp., with EC_50_ values of 56.67 ± 3.78 µg/mL and 89.75 ± 12.75 µg/mL, respectively. Geranylgeraniol also displayed antifungal activity against *Alternaria solani* (*Ell. et Mart.*) *Jones et Grout*, albeit to a lesser extent. The findings of this study provide a scientific foundation for the metabolic engineering of neocembrene and offer novel perspectives on enhancing the nutritional value of rice.

## 1. Introduction

Macrocyclic terpenoids are a class of relatively unstable, structurally unique and diverse natural products composed of 12 or more carbon rings [1]. Most of the reported macrocyclic terpenoids are diterpenes, including conifers, casbenes, neocembrene, jatrophanes and rotaxanes, which are widely distributed in plants and marine organisms and are considered biological genetic precursors of complex polycyclic terpenoids such as taxanes and latvianes [2,3,4]. Diterpenes are compounds with 20 carbon atoms and a molecular framework consisting of four isoprene structures. Diterpenoid compounds include the alkane-type diterpenoid superfamily formed by the initial bicyclization of geranyl diphosphate (GGDP), as well as the casbene-type diterpenes formed by the monocyclic cyclization of GGDP [5].

The Euphorbiaceae plants are well known in ethnic medicine [6]. Analysis of extracts from many species of the Euphorbiaceae family shows that the biological activity of these plants is, in many cases, due to specific diterpenoid compounds [7]. These diterpenoids include the giant hellebarylmethylbutene esters and phorbol derivatives, such as resin toxins and prostaglandins, which are approved for the treatment of precancerous skin diseases (actinic keratosis) and are used in the treatment of severe pain and in HIV research, respectively [8].

Research has shown that castor contains a large number of structurally diverse casbene-type diterpenoids, many of which have pharmacological activity. There are studies reporting that castor oil-type diterpenoids have anticancer cytotoxicity [9,10], anti-inflammatory properties [11,12], anti-HIV [13] and immunosuppressive activity [14], and other effects. At the same time, the casbene-type diterpenoid compound casbene in gramineous rice was reported to be a plant nutrient that has the effect of resisting rice blast disease and bacterial leaf blight [15].

Some casbene-type diterpenes have been obtained by primitive chemical separation, purification, and natural product identification methods as well as heterologous expression biosynthesis. The reported main compounds of casbene-type diterpenoids include casbene, neocembrene and their derivatives: 5-hydroxy-casbene, 5-keto-casbene, 5-keto-7,8-epoxy-casbene, 5-keto-neocembrene, 6-hydroxy-5,9-diketocasbene, jolkinol C, epi-jolkinol C, 6α-hydroxy-10b-methoxy-7a, 8a-epoxy-5-oxocasbane-20,10-olide(3E,6Z,11E)-hydroxy-casba8-3,6,11-trien-5,9-dione, and (3E,7Z,11E)-19-hydroxycasba-3,7,11-trien-5-one [16,17,18].

The species with the most complete genome sequence among Euphorbiaceae plants capable of producing casbene-type diterpenes is *Ricinus communis* L. [17]. Researchers have selected five species of Euphorbiaceae plants to study the biosynthesis of casbene-type diterpenes and reported the distribution of casbene-type diterpenes synthase in this family. Four casbene-type diterpenes synthase genes were expressed in metabolically modified *Saccharomyces cerevisiae*, namely *CAS1* and *CAS3* from *Ricinus communis* L., *TPS10* from *Triadica sebifera* (Linnaeus) Small, and *TPS2* from *Euphorbia lactiflora*. A high-level production of casbene at 31 mg/L was achieved in *S. cerevisiae* [19]. At the same time, researchers discovered casbene-type diterpenes synthase *RcCAS2* from *Ricinus communis* L., which produces neocembrenes, a casbene-type diterpenes. This is the first report of the neocembrene synthase gene [19]. The gene cluster of casbene-type diterpenes biosynthesis genes from the Euphorbiaceae castor family includes the casbene synthase and cytochrome P450 from the *CYP726A* subfamily. *CYP726A14*, *CYP726A17* and *CYP726A18* could catalyze the oxidation of casbene at position 5 to form 5-keto-casbene, a conserved oxidation step in the biosynthesis of important terpenes of the family. *CYP726A16* catalyzes the epoxidation of 5-keto-casbene at positions 7 and 8 to form 5-keto-7,8-epoxy-casbene, while *CYP726A15* catalyzes the oxidation of neocembrene at position 5 to form 5-keto-neocembrene [17]. Evidence for similar gene clusters has also been found in two other species of Euphorbiaceae plants, including *Euphorbia peplus* L., which has a large source of methyl butenoate derived from the treatment of solar keratosis [17]. Three enzymes involved in the cyclization pathway of casbene to jolkinol C (latirane-type diterpene) were identified from mature seeds of *Euphorbia lathyris* L., including two cytochrome P450 enzymes from the CYP71 family and an alcohol dehydrogenase (*ADH*). *CYP71D445* and *CYP726A27* catalyze region-specific oxidation at position 9 and position 5 to form 9-keto-casbene and 5-keto-casbene. In combination with these P450-catalyzed mono-oxidation reactions, the successor ADH1 catalyzes the dehydrogenation of hydroxyl groups, leading to subsequent rearrangement and cyclization [20].

Not only Euphorbiaceae contain casbene-type diterpenes; *Oryza sativa* also contains casbene-type diterpenes. Zhan [15] reported a casbene gene cluster in rice in 2020. The gene cluster on chromosome 7 (DGC7) encodes the entire biosynthetic pathway of 5,10-diketo-casbene, which is catalyzed by OsTPS28 to form casbene from the substrate GGPP, and then continuously modified by *OsCYP71Z21* to form 10-keto-casbene at position 10 and modified 10-keto-casbene at position 5 by *OsCYP71Z25* to form 5,10-diketo-casbene. DGC7 is directly regulated by JMJ705 through epigenetic control mediated by methyl jasmonate. In addition, OsTPS28 can also catalyze the formation of minor neocembrene by the substrate geranyl pyrophosphate (GGPP). In several previously mentioned species of the family Euphorbiaceae, a casbene synthase with a function equivalent to OsTPS28 exists. However, OsTPS28 and casbene synthase evolved independently in Euphorbiaceae species [15].

Casbene and neocembrene are important precursors of casbene-type macrocyclic diterpenes. There is currently a lot of research on multi-species casbene synthase, while there is relatively little in-depth study on neocembrene synthase. The aim of this study is to compare and analyze the reported casbene synthases and neocembrene synthases among multiple species such as Euphorbiaceae and Poaceae, in order to discover more synthetic neocembrene synthases.

Recently, a member of the same research group published an in vitro enzyme activity verification of the OsTPS2 function and an in-depth study of its catalytic mechanism [21]. Therefore, this article focuses on the gene function of OsTPS2 in different hosts such as rice, tobacco, and yeast. This conclusion is the first to report the gene function of OsTPS2 in different species in vivo, which can better verify the hypothesis of in vitro results and facilitate further exploration of the role of OsTPS2 gene overexpression in vivo.

## 2. Results

### 2.1. Comparative Analysis of Candidate Genes for Biosynthesis of Neocembrene

**Figure 1:**
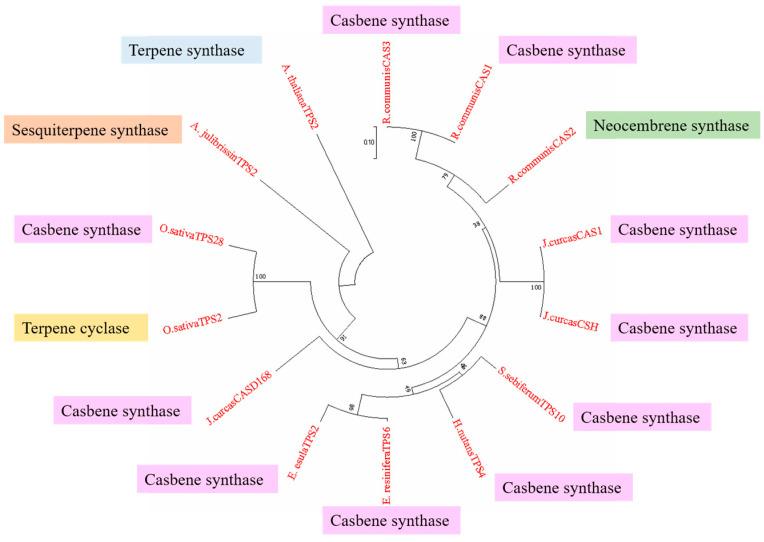
Analysis of the multispecies evolutionary tree of candidate genes for casbene-type diterpene biosynthesis.

**Figure 2:**
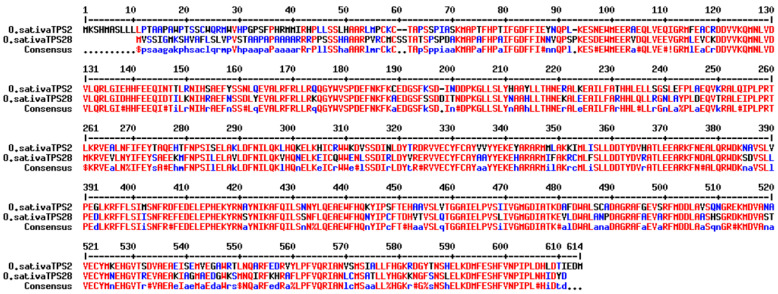
OsTPS2 and OsTPS28 protein sequence comparison. (Red represents the same amino acid, while blue and black represents different amino acids).

This study undertakes a comparative analysis of the casbene and neocembrene synthases reported in various species within the Euphorbiaceae, Poaceae, and other families, with the aim of investigating the potential presence of neocembrene synthase in rice (*Oryza sativa* L.). The analysis encompasses multiple species of *casbenes*, including *Jatropha curcas* L., *Sapium sebiferum*, *Homalanthus nutans*, *Euphorbia esula* L., *Eucalyptus resinifera*, *Arabidopsis thaliana*, and *Albizia julibrissin* Durazz. The phylogenetic tree (Figure 1) and the protein sequence similarities (Table 1) across terpene synthases from different species reveal that *RcCAS1*, *RcCAS2*, and *RcCAS3* exhibit high homology with *JcCAS1*, with a protein similarity exceeding 65%. In contrast, the homology with OsTPS28, a known casbene, and neocembrene synthases in rice, is comparatively low, with protein sequence similarity falling below 35%. Notably, the homology between castor *RcCAS2* and OsTPS2, which has been identified as a terpene cyclase in rice, is greater than that observed with OsTPS28. Further comparison of the protein sequences of OsTPS2 and OsTPS28 indicates a protein sequence similarity of 71.10% (Figure 2), which surpasses the 65% similarity observed among castor proteins *RcCAS1*, *RcCAS2*, *RcCAS3*, and *JcCAS1*. Consequently, this study posits that OsTPS2, characterized as a terpene cyclase, may possess the functional capacity of a casbene-type diterpenoid synthase. However, to date, there have been no reports confirming whether OsTPS2 functions in vivo. Therefore, additional research is warranted to elucidate the gene function of OsTPS2 in yeast, tobacco, and rice and to compare it with the gene function of OsTPS28.

### 2.2. Tobacco Transient Expression Results of *OsTPS2* and *OsTPS28*

**Figure 3:**
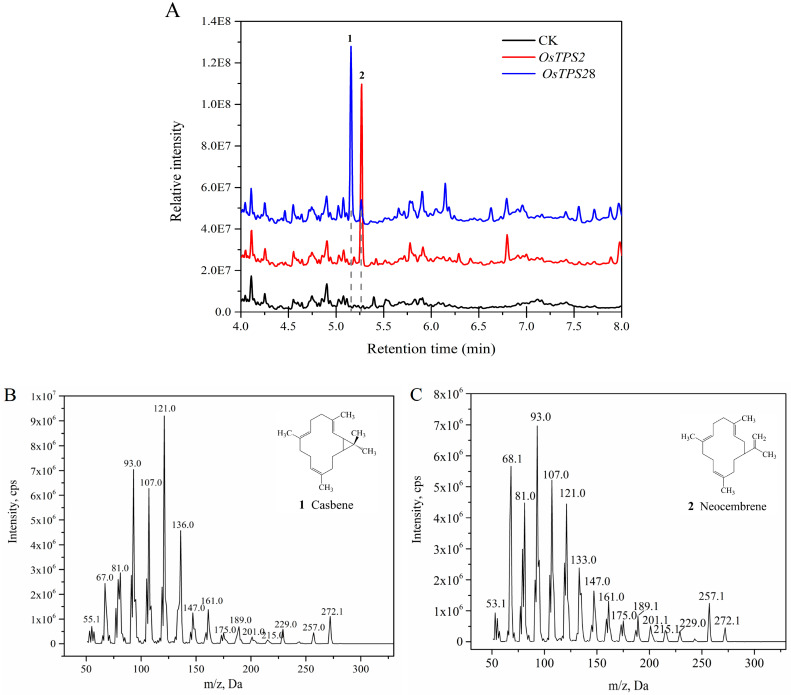
Functional analysis of OsTPS2 and OsTPS28 in the transient expression of tobacco. (**A**) The results of transient tobacco expression, (**B**) the secondary mass spectrometry ion diagram of product **1**, casbene, and (**C**) the secondary mass spectrometry ion diagram of product **2**, neocembrene, in tobacco.

Through the analysis of the experimental results regarding the transient expression of OsTPS2 and OsTPS28 in tobacco (Figure 3, Table S1), it is evident that tobacco plants injected with OsTPS28 predominantly produced the primary product **1**, casbene (RT = 5.158), along with a byproduct **2**, neocembrene (RT = 5.265). In contrast, no casbene was detected in the tobacco injected with OsTPS2. Analysis revealed a singular peak at 5.269 min, identified as neocembrene. Crucially, the retention time and secondary mass spectra of this compound were indistinguishable from those of the byproduct **2** generated by OsTPS28. This congruence confirms that neocembrene was the primary product of OsTPS2. OsTPS28 exhibits a dual synthase function in tobacco, facilitating the synthesis of both casbene and neocembrene, with casbene being the predominant product and neocembrene occurring as a byproduct in lower quantities. Conversely, OsTPS2 is unable to synthesize casbene, with neocembrene serving as its main product. Notably, the transient expression analysis revealed that the quantity of neocembrene synthesized by OsTPS2 was significantly greater than that produced by OsTPS28, approximately 6.4 times higher. This observation suggests that OsTPS2 can also synthesize neocembrene. Further validation of the role of OsTPS2 in neocembrene synthesis will be conducted through yeast-induced expression studies.

### 2.3. Yeast-Induced Expression Results of *OsTPS2* and *OsTPS28*

**Figure 4:**
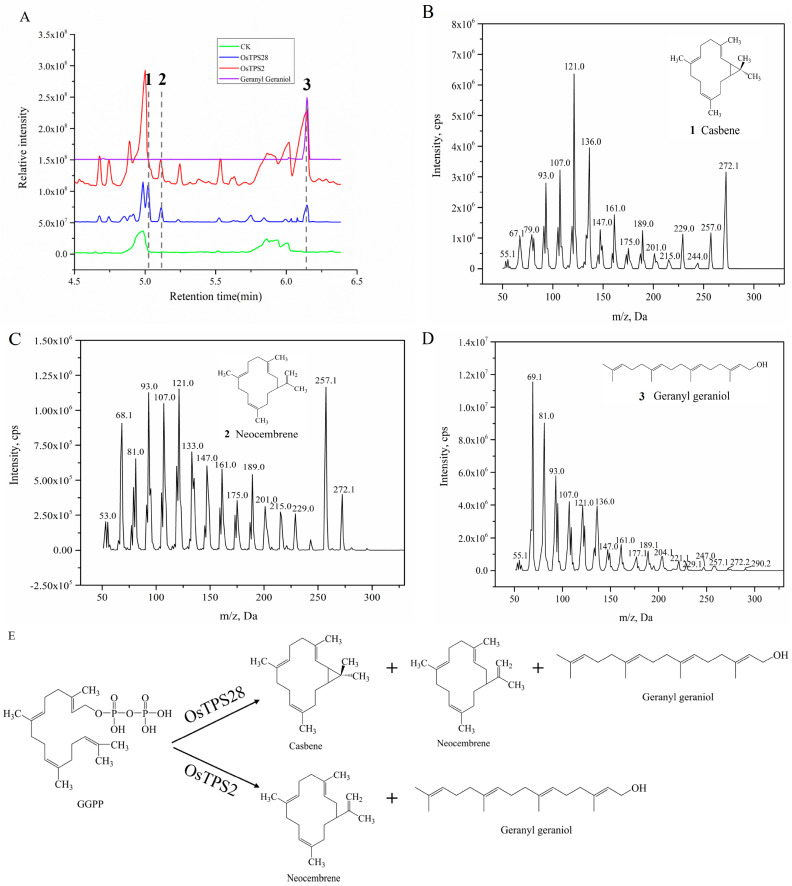
Functional analysis of OsTPS28 and OsTPS2 in yeast-induced expression. (**A**) The result of yeast-induced expression in yeast. (**B**) The secondary mass spectrometry ion diagram of product **1**, casbene, in yeast. (**C**) The secondary mass spectrometry ion diagram of product **2**, neocembrene, and (**D**) the secondary mass spectrometry ion diagram of product **3**, geranylgeraniol, in yeast. (**E**) OsTPS28 and OsTPS2 utilize GGPP substrate to produce different products in yeast-induced expression.

Using GGPP as a substrate, yeast was employed to induce the expression of OsTPS2 and OsTPS28, with their expression products subsequently analyzed via gas chromatography-tandem mass spectrometry (GC-MS/MS). The expression products of OsTPS28 were found to include not only the primary product **1**, casbene (RT = 5.024 min), but also a product **2**, neocembrene (RT = 5.116 min) (Figure 4A–C). In the yeast expression products of OsTPS2, casbene was not detected. However, product **2**, neocembrene, was identified (RT = 5.111 min) (Figure 4A,C, Table S2). Notably, the RT for both products was identical, and the secondary mass spectra for the neocembrene produced by OsTPS2 and OsTPS28 were also consistent (Figure 4C). This finding suggests that OsTPS2 possesses the enzymatic capability to function as a neocembrene synthase, a casbene-type diterpene, which has not been previously reported. Furthermore, it was observed that the concentration of neocembrene synthesized from OsTPS2 was greater than that synthesized from OsTPS28 in the yeast-induced expression products. In addition, it is also possible that yeast endogenous enzymes participate in the process to form geranylgeraniol. However, the hypotheses of spontaneous formation and endogenous enzyme involvement are only hypotheses, and have not yet been validated by actual data.

In addition, product **3** (RT = 6.146) was detected in yeast-induced expression, with both OsTPS2 and OsTPS28 demonstrating the capability to produce this compound. Through standard validation (Figure 4A,D), product **3** was identified as geranylgeraniol. Notably, it was previously reported that the neocembrene synthase *RcCAS2* in castor did not yield product **3**, geranylgeraniol, under yeast-induced expression conditions [19]. Consequently, this study represents the first report indicating that OsTPS2 and OsTPS28 can synthesize geranylgeraniol in yeast. Additionally, it is the first time that OsTPS2 in rice has been identified as a neocembrene synthase (Figure 4A,B,E). This research also provides the first evidence that both OsTPS28 and OsTPS2 can produce geranylgeraniol in yeast-induced expression (Figure 4A,D,E), with the yield of geranylgeraniol synthesized by OsTPS2 being approximately twice that of OsTPS28. Although OsTPS2 and RcCAS2 share similar functions in the synthesis of neocembrene, significant differences exist in their protein sequences. Phylogenetic analysis indicates that OsTPS2 and *RcCAS2* have evolved independently (Figure 1), suggesting that OsTPS2 and *RcCAS2* exemplify a functional “convergent evolution” of genes. Both OsTPS28 and OsTPS2 belong to the TPS-a1 gene family, sharing the same catalytic substrate, GGPP. However, their catalytic products differ. The primary catalytic product of OsTPS28 is casbene, while OsTPS2 exclusively produces neocembrene. Furthermore, this study reveals that the structures of neocembrene and casbene are highly similar (Figure 4E), suggesting that OsTPS28 and OsTPS2 exemplify a functional “divergent evolution” of genes.

### 2.4. Validation of Antifungal Activity of Geranylgeraniol

OsTPS2 and OsTPS28 did not detect product **3** in tobacco. However, both OsTPS2 and OsTPS28 were capable of producing product **3** through yeast-induced expression. Product **3** was confirmed to be geranylgeraniol, a linear diterpenoid compound, by comparison with standard samples. This article posits that the ability of OsTPS2 and OsTPS28 to produce geranylgeraniol in yeast-induced expression may be attributed to their function in removing the diphosphate group from the GGPP structure within the yeast, which subsequently undergoes a spontaneous reaction with water to incorporate hydroxyl groups, resulting in the formation of the linear diterpene geranylgeraniol.

**Figure 5:**
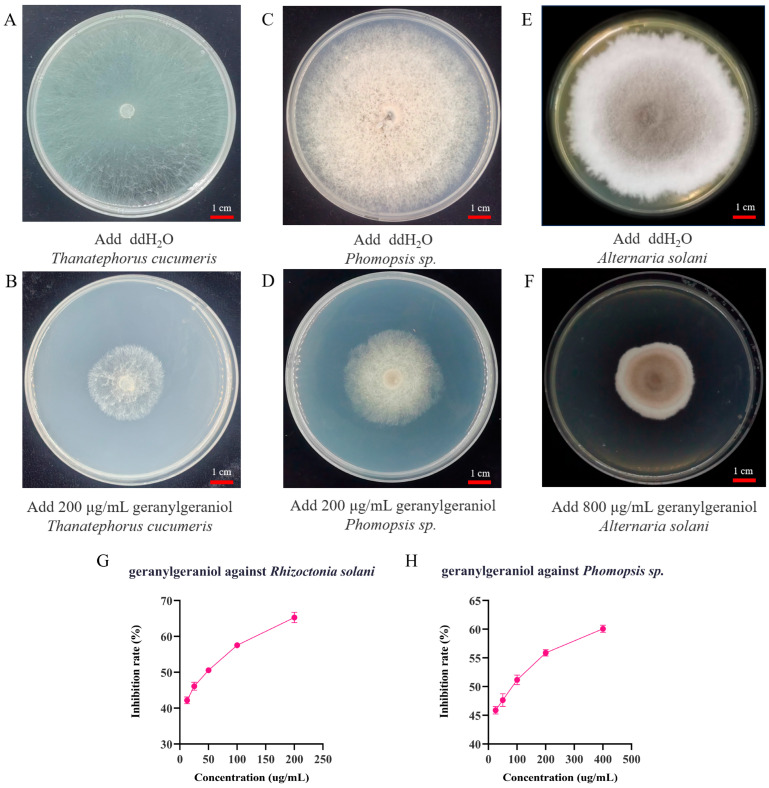
The antifungal activity of geranylgeraniol in vitro. (**A**,**C**,**E**) represent adding only water as a control in three different fungi, while (**B**,**D**,**F**) represent adding 200 μg/mL, 200 μg/mL, and 800 μg/mL of geranylgeraniol to the culture medium, respectively. (**G**,**H**) represent the inhibition rates of *Rhizoctonia solani* and *Phomopsis* sp., respectively.

It has been documented that casbene is a plant preservative exhibiting antibacterial and antifungal properties against rice [15]. However, the antifungal activity of geranylgeraniol, a straight-chain diterpene, against rice diseases within the Poaceae family has not been previously reported. This study aimed to validate the antifungal efficacy of geranylgeraniol against prevalent fungal diseases. The findings indicated that geranylgeraniol (200 μg/mL) exhibited no activity against the rice blast fungus (*Pyricularia oryzae Cav.*). However, it demonstrated antifungal activity against both rice sheath blight (*Rhizoctonia solani*) and *Phomopsis* sp., with effective concentration (EC_50_) values of 56.67 ± 3.78 μg/mL and 89.75 ± 12.75 μg/mL, respectively (Tables S4 and S5). Additionally, geranylgeraniol was found to possess some antifungal activity against *Alternaria solani (Ell. et Mart.) Jones et Grout* (Figure 5).

### 2.5. Overexpression in Rice of *OsTPS2* Detected Neocembrene

**Figure 6:**
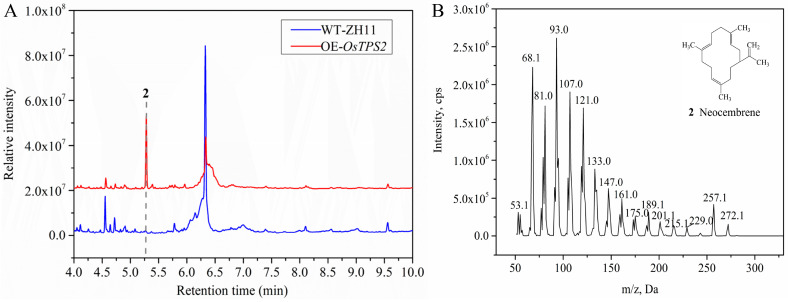
Overexpressed gene function of OsTPS2 in rice. (**A**) The result of overexpressed OsTPS2 in rice of ZH11. (**B**) The secondary mass spectrometry ion diagram of product **2**, neocembrene, in rice.

Following the initial verification of the gene function of OsTPS2 in rice, we proceeded to overexpress OsTPS2 to ascertain its role in the synthesis of neocembrene in rice. A comparative analysis with the control group revealed a significant production of neocembrene (RT *=* 5.279) in the rice leaves overexpressing OsTPS2, whereas neocembrene levels were ten times lower in the control group (Figure 6, Table S3).

**Figure 7:**
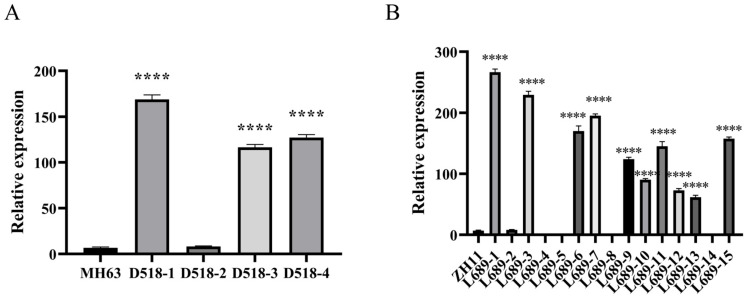
The gene expression level of OsTPS2-OE in MH63 and ZH11. (**A**) The gene expression level of OsTPS2-OE in MH63. D518-1 to D518-4 represent the serial numbers of OsTPS2 overexpression vectors. (**B**) The gene expression level of OsTPS2-OE in ZH11. L689-1 to L689-15 represent the serial numbers of OsTPS2 overexpression vectors. Stars represent significance, and four stars represent extreme significance.

Meanwhile, comparative analysis between the wild-type (WT) controls and OsTPS2-overexpressing (OsTPS2-OE) lines revealed distinct expression patterns in the two genetic backgrounds. In the MH63 background, three out of four transgenic lines (D518-1, D518-3, and D518-4) exhibited a substantial increase in OsTPS2 expression, ranging from 17- to 25-fold higher than that in the WT control, whereas line D518-2 showed no significant difference compared to the WT. Similarly, in the ZH11 background, ten out of fifteen independent lines demonstrated successful overexpression, with expression levels 8- to 38-fold higher than those in the WT. However, four lines failed to overexpress the gene, and one line showed no significant change relative to the WT (Figure 7, Tables S6 and S7).

This finding further substantiates the conclusion of this study that OsTPS2 functions as a neocembrene synthase. However, it is noteworthy that the product geranylgeraniol was not detected in either the rice overexpressing OsTPS2 or the control group.

## 3. Discussion

### 3.1. Gene Function Mining of *OsTPS2*

Through a synthesis of prior research findings, it has been determined that the gene function of *TPS2* exhibits variability across different species. For instance, the *EeTPS2* gene from the Euphorbiaceae family is known to facilitate the synthesis of casbene. However, it has not been documented to synthesize neocembrene [19]. In contrast, the *AjTPS2* gene from Acacia, a member of the Fabaceae family, is responsible for the synthesis of sesquiterpene synthase [22], while *AtTPS2* from Arabidopsis is involved in the synthesis of terpene synthase [23]. Additionally, our research group [15] found that in Poaceae rice, the genes OsTPS2 and OsTPS28 exhibit similar genetic distances on chromosomes 7 and 1, respectively, and demonstrate a high degree of homology within rice. However, the gene function of OsTPS2 is not discussed in the literature, as OsTPS2 and OsTPS28 are not located within the same gene cluster that modifies the cassette. Nevertheless, the article posits that OsTPS2 may function as a terpene synthase, although its specific gene function remains unreported [15]. Consequently, building upon the previous research conducted by our group, this article investigates the rice gene OsTPS2, which has been annotated as a terpene cyclase in the NCBI database. This investigation involves an analysis of the protein sequence similarity among various species of castor oil-type diterpenoid synthases, alongside the prior findings regarding the rice bifunctional synthase OsTPS28. Notably, OsTPS2 shares 71% protein sequence similarity with OsTPS28. Furthermore, the ricin *RcCAS2* and *JcCAS1* genes, which also synthesize casbene-type diterpenes [19], exhibit a protein sequence similarity of 65.23%, which is lower than that observed between OsTPS28 and OsTPS2. It is therefore hypothesized that OsTPS2 may possess a similar function to OsTPS28 in the synthesis of casbene-type diterpenes [21].

### 3.2. Comparison of Synthetic Products of *OsTPS2* and *OsTPS28*

In this study, yeast was employed to induce the expression of OsTPS2 and OsTPS28. A comparative analysis of the expression products revealed that OsTPS2 is capable of catalyzing the substrate geranylgeranyl pyrophosphate (GGPP) to produce neocembrene, while it does not facilitate the production of casbene. The functional role of OsTPS2 aligns with the previously documented function of *RcCAS2* in castor [19]. The identification of the gene function of OsTPS2 in synthesizing neocembrene represents the first report concerning this process in Poaceae rice. Conversely, OsTPS28 not only generates the previously reported casbene but also neocembrene, corroborating the findings [15]. Due to the absence of standard samples for casbene and neocembrene in this investigation, the secondary mass spectra of products **1** and **2** were compared with the corresponding compounds in the National Institute of Standards and Technology (NIST) database for gas chromatography-mass spectrometry (GC-MS), revealing consistency with the secondary mass spectra of casbene and neocembrene documented in prior literature [15,19]. Unfortunately, it is not possible to purchase standard samples of casbene and neocembrene for accurate verification. Consequently, it was determined that product **1** is casbene and product **2** is neocembrene. Additionally, both OsTPS2 and OsTPS28 are capable of cleaving the diphosphate group from GGPP to yield product **3**, a linear diterpene known as geranylgeraniol. Following the removal of the diphosphate group from GGPP by OsTPS2 and OsTPS28, the hydroxyl groups may be formed spontaneously through non-enzymatic reactions with water, which is consistent with previously reported findings regarding hydroxyl groups generated through such non-enzymatic processes [16]. However, there is no existing literature indicating that *RcCAS2* produces geranylgeraniol through yeast-induced expression [19], and the biosynthetic pathway for geranylgeraniol has yet to be elucidated. Therefore, the induction of geranylgeraniol production in yeast by OsTPS2 and OsTPS28 constitutes another novel finding presented in this article. This study posits that OsTPS2 and OsTPS28 represent instances of “divergent evolution”, the same substrate yielding different products, with neocembrene and casbene exhibiting highly similar chemical structures. It is hypothesized that OsTPS2 may have evolved into OsTPS28, with the expression product transitioning from neocembrene as the primary product to casbene as the main product, while neocembrene becomes a byproduct, thereby providing additional precursor substances for the subsequent synthesis of complex macrocyclic diterpenes.

In addition, it was observed that the quantity of neocembrene synthesized by OsTPS2 was significantly greater than that produced by OsTPS28 in tobacco transient expression assays, with an approximate increase of 6.4 times. Concurrently, the geranylgeraniol induced by the expression of OsTPS2 in yeast was also approximately twice as high as that generated by OsTPS28. Consequently, for the purpose of obtaining a substantial yield of neocembrene to facilitate the extraction of high-purity macrocyclic diterpene precursor metabolites, or for the exclusive production of the straight-chain diterpene geranylgeraniol, the expression of OsTPS2 is more advantageous compared to OsTPS28. Furthermore, analysis of the evolutionary tree revealed that both OsTPS2 and *RcCAS2* function as neocembrene synthases. However, they have evolved independently, exhibiting a protein sequence similarity of only 32.14%. This suggests that OsTPS2 and *RcCAS2* represent instances of “convergent evolution”, the same catalytic substrate and product.

### 3.3. The Antifungal Activity of Geranylgeraniol

It has been documented that the primary product of OsTPS28, casbene, exhibits antibacterial properties against rice blast and bacterial blight. However, the antibacterial activity of neocembrene remains unreported, and the lack of available neocembrene standard products precludes further investigation into its antibacterial properties. Nonetheless, both OsTPS2 and OsTPS28 are capable of synthesizing the linear diterpene geranylgeraniol through yeast-induced expression. Geranylgeraniol has been associated with various biological activities, including antibacterial [24], anti-inflammatory [25,26], and anti-tumor [27,28,29], as well as neuroprotective effects [30,31] and the ability to elevate testosterone levels in mice [32]. However, there is a lack of reports regarding the antifungal activity of geranylgeraniol in plant systems, prompting this article to further investigate its antifungal properties. Upon examination of its antifungal activity, it was determined that geranylgeraniol demonstrates significant antifungal efficacy against *Rhizoctonia solani* and *Phomopsis* sp., with EC_50_ values of 56.67 ± 3.78 µg/mL and 89.75 ± 12.75 µg/mL, respectively. The antifungal effect of geranylgeraniol on *Rhizoctonia solani* is lower than casbene-type diterpene phytocassane E, which has an EC_50_ value of 6 μg/mL against *Magnaporthe grisea* in rice [33]. Due to the limited antifungal activity of geranylgeraniol against *Alternaria solani* (*Ell. et Mart.*) *Jones et Grout*, the EC_50_ value for this pathogen will not be calculated. Furthermore, the absence of in vitro fungal inhibition studies on casbene [15] prevents a comparative analysis of the in vitro antifungal activities of geranylgeraniol and casbene. Additionally, no production of geranylgeraniol was detected in both the ZH11 control group and the OsTPS2 overexpression group in rice, thereby hindering the verification of the in vivo antifungal activity of geranylgeraniol in this plant species.

### 3.4. Future Research Expectations

Currently, there are no documented findings indicating that OsTPS2 is involved in the synthesis of neocembrene, nor have there been any reports of OsTPS2 and OsTPS28 facilitating the expression of geranylgeraniol in yeast. Consequently, we plan to validate the in vivo antifungal properties of neocembrene by overexpressing OsTPS2 in rice. Additionally, we aim to generate a substantial quantity of neocembrene through the transient expression of OsTPS2 in tobacco. The extracted and purified neocembrene will then be utilized for subsequent in vitro studies on antibacterial activity.

## 4. Materials and Methods

### 4.1. Construction of *OsTPS2* and *OsTPS28* Vectors

Rice gene cloning method: Extract RNA from Zhonghua 11 rice leaves and reverse transcribe into cDNA as a gene template. The OsTPS2 (*LOC_Os01g42610*) and OsTPS28 (*LOC_Os07g11790*) were cloned using the high-fidelity DNA polymerase KOD Fx (TOYOBO, Shanghai) in a 20 μL system (KOD-Fx buffer: 10 μL, ddH_2_O: 4.1 μL, dNTPs: 4 μL, forward/reverse primer: 0.4/0.4 μL, DMSO: 0.4 μL, KOD Fx: 0.4 μL, cDNA template: 0.3 μL). The PCR reaction program used for KOD-Fx polymerase: predenaturation at 94 °C for 2 min; denaturation at 98 °C for 20 s; annealing at 58 °C for 20 s and extension at 68 °C for 2 min (29 cycles); supplement at 68 °C for 10 min and store at 25 °C for 1 min.

Tobacco vector construction: Join the target gene fragment with the linear vector pEAQ-HT-TPS2/pEAQ-HT-TPS28 by the homologous recombination method (5 μL system: 1 μL target gene fragment, 1 μL linear vector pEAQ-HT-TPS2/pEAQ-HT-TPS28, 1 μL homologous recombinase buffer, 0.5 μL homologous recombinase, 1.5 μL ddH_2_O), incubate for 1 h at 37 °C and immediately cool in ice to obtain a homologous recombinant product. 

Tobacco vector transformation: Transfer the constructed tobacco vector into Agrobacterium GV3101 (WEIDI, Shanghai, China) (containing the P19 protein GV3101 (pSoup-p19)). Take the Agrobacterium suspension stored at −80 °C and melt it at room temperature (immediately place it on ice after melting). Take out 15 μL of the agrobacterium suspension and transfer it to a sterilized EP tube for conversion preparation. Take 100 ng of plasmids and add them to the agrobacterium competence cells. Mix gently with a pipette and let stand on ice for 5 min. Then place it in liquid nitrogen for 5 min. Next, remove the EP tube from the liquid nitrogen and place it in a water bath at a constant temperature of 37 °C for 5 min. Finally, put it back on ice for 5 min. Add 100 μL of LB liquid medium and incubate for 1 h at 28 °C in a shaker. Distribute 100 μL of bacterial solution evenly into LB plates containing antibiotics, invert, and incubate at 28 °C for 2–3 days. After the bacteria have grown, identify the bacterial colonies using PCR and subject the positive agrobacterium to subsequent infection experiments.

Yeast vector construction: The target gene fragment was ligated with the linear vector pESC-URA-TPS2 using the homologous recombination method (5 μL system: 1 μL target gene fragment, 1 μL linear vector pESC-URA-TPS2, 1 μL homologous recombinase buffer, 0.5 μL homologous recombinase, 1.5 μL ddH_2_O). After incubation at 37 °C for 1 h, the homologous recombinant product was immediately cooled in ice to obtain the homologous recombinant product.

Yeast transformation method: Add pESC-URA-TPS2 or pESC-URA-TPS28 plasmid (100–500 ng) to 10 μL of yeast competent cells, then add EZ3 solution (Zymo Research, Irvine, CA, USA) to the yeast transformation kit, mix thoroughly and place in a 30 °C dry bath for 45 min. Mix thoroughly every 15 min, repeat three times and then transfer the transformed yeast to a prepared solid yeast culture medium with defects. Incubate at 28 °C for 2–3 days. After a single bacterium has been cultured, a few colonies can be collected for PCR validation.

### 4.2. Transient Expression Methods of *OsTPS2* and *OsTPS28* in Tobacco

The receptor for the immediate transformation experiment is *Nicotiana tabacum*, which had been growing for 5–6 weeks. Cultivation takes place in an environment with a temperature of 25 °C, 65% humidity and 14 h of light/10 h of darkness. Place positive Agrobacterium colonies in 500 μL of LB medium with antibiotics and culture for 20–24 h. Add 200 μL of the cultured bacterial solution to 5 mL of LB medium with antibiotics, and incubate at 28 °C in a shaker at 220 rpm until OD600 = 2.0. Then centrifuge at 10,000 rpm for 2 min at room temperature to collect the bacteria, and then wash and collect the bacterial cells with ddH_2_O. Resuspend the bacterial cells in infection solution (containing 10 mmol MgCl_2_, 10 mmol MES, 150 μmol acetyl-eugenol, pH = 5.6) until OD600 = 1.0, and incubate for 2–3 h at room temperature in the dark. Use a 1 mL syringe with the needle removed to draw up an appropriate amount of bacterial solution. Place your finger on the front of the tobacco leaf and allow the bacterial solution to penetrate from the back of the leaf. Use a marker pen to circle the water-stained area of the tobacco leaf. Following infiltration, the tobacco plants were maintained under standard greenhouse conditions, and leaf samples were harvested 3–5 days post-inoculation (dpi) for subsequent analysis.

### 4.3. Yeast-Induced Expression Methods for *OsTPS2* and *OsTPS28*

Yeast-induced expression method: After sterilizing with yeast uracil-deficient medium (8 g/L) (FunGenome, Beijing, China), 250 mL of the medium was transferred into a sterile triangular flask. An appropriate volume of 40% sterile glucose solution was then added to achieve a final concentration of 40% glucose at a ratio of 95:5 (*v/v*). Pre-transformed yeast colonies were introduced into the culture medium and shaken in a shaker for 2–3 days at 28 °C and 220 rpm. The shaken yeast was centrifuged under sterile conditions, and the supernatant was discarded. The yeast pellet was suspended and centrifuged with sterile double-distilled water, repeating this process twice to thoroughly wash away the glucose solution. The resulting yeast was then transferred to a new sterile binary-deficient medium, to which an appropriate volume of 20% sterile lactose solution was added at a ratio of 90:10 (*v/v*). Expression was induced at 30 °C and 150 rpm for 72 h, followed by the collection and extraction of metabolites.

### 4.4. Construction and Transformation of Rice Overexpression Materials

The vectors associated with genetic transformation in rice were constructed utilizing the Gateway method, employing pDONR207 as the entry vector and pJC034 as the overexpression vector. This study implemented the Agrobacterium-mediated genetic transformation technique to acquire transgenic materials. The Agrobacterium strain utilized was EHA105, while the ZH11 variety served as the recipient material for the genetic transformation. The transformation methodology was adapted from the work [34]. Mature ZH11 seeds from recent harvests should be selected and the seed coat removed. Initially, the seeds must be treated with 75% ethanol for one min, followed by disinfection in a 0.15% HgCl_2_ solution for 15 min. Subsequently, the seeds should be washed with sterile water eight times. The disinfected seeds are then cultured in a dark medium for a duration of 30 to 40 days at a temperature of 26 °C to induce callus formation. The resulting callus can be subcultured twice, with a 15-day interval between each subculture. The callus on the subculture medium should be immersed in a prepared Agrobacterium solution for 30 min, after which it is cultured at 19 °C for three days under dark conditions. Following co-cultivation, the callus must be washed eight times with a substantial volume of sterile water to eliminate any residual *Agrobacterium*. The callus tissue is then transferred to a culture dish containing resistance hormones to promote the development of a resistant callus. Once the resistant callus is obtained, it should be transferred to differentiation medium and allowed to differentiate and grow into seedlings. The differentiated seedlings should be carefully extracted, refined, and subsequently transferred to the field for cultivation.

### 4.5. Extraction and Detection Methods of Metabolites

Referring to methods in earlier works [35], the metabolic samples derived from tobacco or rice leaves were initially subjected to drying in a vacuum freeze dryer for a duration of seven days to eliminate tissue moisture. Subsequently, the dried samples were ground into a fine powder using liquid nitrogen. A precise weight of 0.1 g of the powdered material was measured and placed into separate 2 mL centrifuge tubes. To each tube, 1 mL of n-hexane extract was added, followed by vortexing three times in a vortex shaker, with each vortexing session lasting 30 s and occurring every 10 min. The samples were then stored in a refrigerator at 4 °C for a period of 16 h to facilitate the extraction of substituted metabolites at low temperatures. Following this incubation, the samples were vortexed again for 30 s and subsequently placed in a pre-cooled freeze centrifuge at 4 °C. The centrifuge lid was securely closed, and the samples were centrifuged for 10 min at 10,000 rpm. The precipitate was discarded, and the liquid phase was extracted. Finally, the supernatant was filtered through a 0.22 μm organic phase nylon filter membrane into a centrifuge tube, resulting in the preparation of the final GC-MS/MS metabolic sample for machine detection.

Following yeast induction expression, the bacterial cells were harvested via centrifugation. The strain was subsequently frozen in liquid nitrogen and ground into a fine powder. The bacterial powder was then subjected to extraction using 100 mL of n-hexane, with metabolites being extracted by shaking the mixture at 37 °C and 220 rpm for a duration of two days. The resulting bacterial powder was filtered to isolate the extract, which was subsequently concentrated by removing n-hexane using a vacuum rotary evaporator to yield the extracted product. The final extract was dissolved in n-hexane at an appropriate concentration to prepare samples for GC-MS/MS analysis, followed by filtration and preparation for detection.

The gas chromatography parameters employed in this study involved the use of an Agilent GC-MS/MS-7890 system (Santa Clara, CA, USA). The chromatographic column utilized was a SHIMADZU SHRx-5MS model (Chengdu, China), measuring 30 m in length, with an internal diameter of 0.25 mm and a film thickness of 0.25 µm. The injection port temperature was maintained at 270 °C. The initial temperature of the column was set at 80 °C, which was subsequently increased to 300 °C at a rate of 15 °C per minute. The temperature of the fourth stage rod in the mass spectrometer was set to 150 °C. The collision gas used was high-purity nitrogen at a flow rate of 1.5 mL/min, while the quenching gas was high-purity helium at a flow rate of 2.25 mL/min. A liquid injection mode without splitting was adopted, with an injection volume of 2 µL.

### 4.6. Antifungal Experimental Methods of Geranylgeraniol

Preparation of 1 L of potato dextrose agar (PDA) culture medium: Add 200 g of potatoes to 1 L of ddH_2_O and boil in water at 100 °C until the potatoes are soft and tender. Filter the mixture to retain the potato water, then adjust the volume to 1 L. Divide the potato water into 60 mL portions and transfer each portion into a triangular flask. Add 1.2 g of glucose and 1.08 g of agar powder. Seal the flasks with a sealing membrane and sterilize at 121 °C for 15 min to obtain the PDA culture medium.

Preparation of geranylgeraniol concentration: Prepare a geranylgeraniol mother liquor using anhydrous ethanol at a concentration of 80,000 μg/mL. Subsequently, dilute this solution with ddH_2_O for six concentration gradients. The final concentrations after adding 60 mL of culture medium will be 12.5 μg/mL, 25 μg/mL, 50 μg/mL, 100 μg/mL, 200 μg/mL, 400 μg/mL and 800 μg/mL. 

Detection of antifungal activity using the inhibition zone method: Different concentrations of geranylgeraniol solution were added to the culture medium, mixed thoroughly, and poured into 9 cm culture dishes. A total of 60 mL of culture medium was evenly distributed among three culture dishes, referred to as A_i_, and ddH_2_O was used as the control, labeled A_0_. Once the culture medium solidified, a circular fungal disk with a radius of approximately 0.5 cm was placed on top and inverted into the culture medium. The culture dish was then sealed and incubated upside down in an incubator set at 28 °C for 3 to 5 days. After the control group was established, the cross method was employed to measure the inhibition zone, and photographs of the phenotypes in each culture dish were taken. 

The calculation formula for the inhibition rate is expressed as follows: inhibition rate = (A_0_ − 0.5) − (A_i_ − 0.5)/(A_0_ − 0.5) × 100%, where A_0_ and A_i_ are the average of three replicates.

### 4.7. Statistical Analysis

In each experiment, the results are presented as the mean ± standard deviation (SD) derived from three biological replicates. One-way analysis of variance (ANOVA) was performed using SPSS 21.0 software. The significance level was set at *p* < 0.05. Data and image analyses were conducted utilizing software programs including Origin Pro 9.0, ChemDraw 2019, and Adobe Illustrator. Additionally, evolutionary tree analysis was performed using MEGA 7 software, https://espript.ibcp.fr/ESPript/ESPript/ (accessed on 18 December 2023), for protein sequence alignment analysis.

## 5. Conclusions

After conducting a comparative analysis of the evolutionary trees and protein sequences of various species of casbene synthase and neocembrene synthase, this study has identified and confirmed that OsTPS2 from the Poaceae family, specifically rice, possesses the genetic capability to synthesize neocembrene, as demonstrated through both tobacco transient expression and yeast-induced expression assays. Furthermore, it was established that OsTPS28 is capable of synthesizing not only the primary product casbene, but also the by-product, neocembrene. Notably, the synthesis of neocembrene by OsTPS2 was found to be at a higher concentration compared to that produced by OsTPS28. Additionally, the overexpression of OsTPS2 in rice resulted in an increased concentration of neocembrene in the leaves of the rice plants. Both OsTPS2 and OsTPS28 were also shown to induce the synthesis of the straight-chain diterpene geranylgeraniol in yeast, which exhibits antibacterial properties against *Rhizoctonia solani* and *Phomopsis* sp., with EC_50_ values of 56.67 ± 3.78 μg/mL and 89.75 ± 12.75 μg/mL, respectively. Moreover, geranylgeraniol demonstrated some antibacterial activity against *Alternaria solani* (*Ell. et Mart.*) *Jones et Grout*. The findings of this study offer new insights into the biosynthesis of casbene-type diterpenes in rice and linear diterpenes in yeast.

## Figures and Tables

**Table 1:** Comparison of the sequence similarity of casbene-type diterpene synthase proteins.

Gene Names	Gene Functions	Gene Names	Gene Functions	Protein Sequence Similarity
*RcCAS1*	Casbene synthase	*RcCAS3*	Casbene synthase	95.01%
*RcCAS1*	Casbene synthase	*RcCAS2*	Neocembrene synthase	73.84%
*RcCAS3*	Casbene synthase	*RcCAS2*	Neocembrene synthase	73.61%
*JcCAS1*	Casbene synthase	*RcCAS1*	Casbene synthase	65.40%
*JcCAS1*	Casbene synthase	*RcCAS3*	Casbene synthase	65.40%
*JcCAS1*	Casbene synthase	*RcCAS2*	Neocembrene synthase	65.23%
OsTPS28	Casbene synthase	*RcCAS1*	Casbene synthase	34.15%
OsTPS28	Casbene synthase	*RcCAS2*	Neocembrene synthase	31.75%
OsTPS28	Casbene synthase	*RcCAS3*	Casbene synthase	34.90%
OsTPS28	Casbene synthase	OsTPS2	Terpene cyclase	71.10%
OsTPS2	Terpene cyclase	*RcCAS1*	Casbene synthase	32.98%
OsTPS2	Terpene cyclase	*RcCAS2*	Neocembrene synthase	32.14%
OsTPS2	Terpene cyclase	*RcCAS3*	Casbene synthase	33.77%
*RcCAS2*	Neocembrene synthase	*AtTPS2*	Terpene synthase	ND
OsTPS2	Terpene cyclase	*AtTPS2*	Terpene synthase	ND

Note: ND indicates no similarity in protein sequence.

## Data Availability

The original contributions presented in this study are included in the article. Further inquiries can be directed to the corresponding authors.

## Supplementary Materials

The following supporting information can be downloaded at: https://www.mdpi.com/article/10.3390/plants15111614/s1, Table S1: Transient expression data of tobacco OsTPS2 and OsTPS28. Table S2: Yeast induced expression date of OsTPS2 and OsTPS28. Table S3: Overexpression in rice date of OsTPS2. Table S4: Geranylgeraniol against Rhizoctonia solani. Table S5: Geranylgeraniol against Phomopsis sp. Table S6: Gene expression level of OsTPS2-OE in MH63. Table S7: Gene expression level of OsTPS2-OE in ZH11.
